# Transcriptome Analysis of Juvenile Tilapia (*Oreochromis niloticus*) Blood, Fed With Different Concentrations of Resveratrol

**DOI:** 10.3389/fphys.2020.600730

**Published:** 2020-12-09

**Authors:** Yao Zheng, Wei Wu, Gengdong Hu, Liping Qiu, Jiazhang Chen

**Affiliations:** ^1^Chinese Academy of Fishery Sciences, Freshwater Fisheries Research Center, Wuxi, China; ^2^Fishery Eco-Environment Monitoring Center of Lower Reaches of Yangtze River, Ministry of Agriculture, Wuxi, China; ^3^Laboratory of Quality & Safety Risk Assessment for Aquatic Products on Environmental Factors (Wuxi), Ministry of Agriculture, Wuxi, China; ^4^Key Laboratory of Control of Quality and Safety for Aquatic Products, Ministry of Agriculture, Wuxi, China

**Keywords:** resveratrol, GIFT tilapia, RNA sequencing, KEGG, DEGs

## Abstract

*Oreochromis niloticus* (genetically improved farmed tilapia, GIFT) often bites the root of *Polygonum cuspidatum* when it is used as a floating bed, and resveratrol (RES) is mainly accumulated in the root of *P. cuspidatum*. Blood acts as a pipeline for the fish immune system. Generating blood transcriptomic resources is crucial for understanding molecular mechanisms underlying blood immune responses. In this study, we determined the effects of RES administration on blood transcriptomic response in GIFT. With increasing RES concentration, 133 (0.025 vs. 0.05 g/kg RES), 155 (0.025 vs. 0.1 g/kg RES), and 123 (0.05 vs. 0.1 g/kg RES) genes were detected as significant differentially expressed genes (DEGs). Three and ninety-five shared significant DEGs were found to be enriched among the three (except 0.1 g/kg RES) and four groups (0, 0.025, 0.05, and 0.1 g/kg RES), respectively. To determine the relationship between mitochondrial regulation and RES supplementation, the results of RNA-Seq were analyzed and nine mitochondria-related genes (ATP synthase or mitochondrial-function-related genes) were verified. The results revealed the same expression pattern: cytochrome c isoform X2 (*cox2*), katanin p60 ATPase-containing subunit A1 isoform X1 (*katna1*), plasma membrane calcium-transporting ATPase 1-like (*atp2b1*) and GTP-binding protein A-like (*gtpbpal*) showed the highest expression in the 0.1 g/kg RES group, while NADH dehydrogenase [ubiquinone] iron-sulfur protein 2 mitochondrial (*nad7*), ATP synthase subunit beta, mitochondrial (*atpb*), ATP synthase subunit alpha, mitochondrial-like (*atpal*), ATP synthase subunit alpha, mitochondrial (*atpa*) and ATP-dependent Clp protease proteolytic subunit, mitochondrial (*clpp*) revealed a dose-dependent expression following RES supplementation. Blood Ca^2+^-ATPase activity, and malondialdehyde, glutathione, and ATP content were significantly increased in the 0.05 (except Ca^2+^-ATPase activity), 0.1 g/kg RES group when compared with the controls. Eighty-nine shared DGEs were mainly enriched in antigen processing and presentation, cell adhesion molecules and phagosome pathways, based on the comparison between previous reported hepatic and the present blood transcriptome. Our study demonstrated that RES supplementation might improve the resistance to metabolism dysfunction via mitochondrial energy synthesis and/or the respiratory chain (e.g., ATPase).

## Introduction

Resveratrol (RES), a polyphenolic phytoalexin, found in numerous plants, particularly in the skin of grapes, has been reported to possess anti-oxidative (Wilson et al., 2015), anti-angiogenic, vascular-targeting, anti-obesogenic, and anti-aging properties (Liu et al., 2015) in several fish models. Although effectual studies have been performed on the mode of action of RES, only a few relevant signal pathways have been identified, such as the oxygen (Wang L. et al., 2016), cytokine (Zheng et al., 2017b), apoptosis (Taylor et al., 2001), and DNA damage pathway (Wang et al., 2015). To select the target gene for verifying the effective pathway, researchers have performed gene cloning (Zhan et al., 2015), transcriptome analysis (Wang L. et al., 2016; Wang Z. et al., 2016; Zhu et al., 2017), and miR-Seq (Qiang et al., 2017) in the corresponding organs, such as the liver (Zheng et al., 2017b), fin, kidney (Sutherland et al., 2014), spleen (Krasnov et al., 2013), and blood.

In the blood, macrophages play an important role in immunogenic challenges associated with reactive oxygen species and pro-inflammatory cytokines upon RES supplementation (Pallarès et al., 2012). The blood immune parameters (Van Doan et al., 2017) and plasma enzyme activities (Gabriel et al., 2015; Zheng et al., 2017a) have been used as endpoints of streptococcosis in tilapia. Studies have showed that floating bed cultivation of the Chinese medical herb *Polygonum cuspidatum* in *Oreochromis niloticus* (genetically improved farmed tilapia, GIFT) ponds not only improves the water quality, but also enhances fish resistance to streptococcal infection (Zheng et al., 2017a). GIFT often bites the root of *P. cuspidatum*, when it is used as a floating bed, and RES is mainly accumulated in the roots of *P. cuspidatum*. To fully use this Chinese medical herb, RES is added to fish feed. It has been reported that 0.025, 0.05, and 0.1 g/kg dietary supplementation of RES induced hepatic inflammatory response (Zheng et al., 2017b). When RES was used as a functional immunopotentiator, it affected the innate immunity of fish (Zhang et al., 2014), and this phenomena has been further verified in tilapia liver of our recent study (Zheng et al., 2019b). Blood acts as a pipeline for the fish immune system, whether the affected pathways is different from the liver, and however, the underlying mechanisms in the blood and differences between the blood transcriptome and previous reported liver transcriptome (Zheng et al., 2017a) have not been determined.

RES has been shown to potentially target several mitochondrial metabolic pathways (Bastin and Djouadi, 2016; Jardim et al., 2017). It can regulate mitochondrial redox status, which in some cases prevents or delays disease progression (Teixeira et al., 2019). RES can reduce the level of autophagy in the apoptosis (apoptosis pathway changed in the streptococcosis affected tilapia, Zhu et al., 2017) process by preventing the accumulation of reactive oxygen species in fibroblast-like synovial cells, which might result in mitochondrial dysfunction (Cao et al., 2018). It has the potential to be used as an immunopotentiator in fish cultivation to determine the relationship between mitochondrial dysfunction and pathogenic prevention. Cytokine-like factors in the blood of Streptococcus-infected tilapia enhance the cytotoxicity of non-specific cells by stimulating gene expression (Evans et al., 2000). Although some target genes have been discovered, information on blood transcriptomic biomarkers (Redei et al., 2014) associated with disease prevention pathways (Ndong et al., 2007) and mitochondrial dysfunction (Nevalainen et al., 2016) is limited. Transcripts of the canonical pathway of mitochondrial dysfunction, particularly related to oxidative phosphorylation, can be considered regulators of several mitochondrial respiratory chain components, resulting in the production of chemical energy.

In the present study, the blood transcriptome of GIFT following RES administration (0, 0.025, 0.05, and 0.1 g/kg) was characterized using the Illumina/Hiseq-2500 RNA-Seq technology. The aims of this study were: (1) to find whether the mitochondrial dysfunction happened in RES-fed *O. niloticus*, the different affected pathways between blood (whether target mitochondrial metabolic pathways) and liver (target immune system pathways in our previous study), and (2) to explain why RES could be used as functional immunopotentiator, for aquatic feed production and disease prevention.

## Materials and Methods

### Collection of Animals

Pretreatment and culture condition: Fertilized eggs of GIFT *O. niloticus* were obtained from the Freshwater Fisheries Research Center of the Chinese Academy of Fishery Sciences, Yixing, China. The fish fry were cultivated in a pond (20 m × 30 m), and acclimatized for 2 weeks before the experiment. Two-month-old *O. niloticus* juveniles were used in the experiment, and they were acclimatized in the aquarium facility with dechlorinated tap water at 25 ± 1°C and 14:10 h light/dark cycle. Throughout the experimental period, water samples were obtained before and after each water change, and the experimental conditions were as follows: pH, 7.0 ± 0.2; dissolved oxygen (tested using YSI 556MPS, United States), 7.42 ± 0.08 mg/L; total phosphate, 2.37 ± 0.05 mg/L; total nitrogen and ammonia nitrogen (by Nessler’s reagent spectrophotometry), 0.48 ± 0.06 mg/L and 0.40 ± 0.08 mg/L, respectively; and total water hardness (ICP-OES, Optima 7000, PerkinElmer, United States), 206.7 ± 4.5 mg/L CaCO_3_.

Feeding trial and concentration selection: The concentration of this study was determined using the method employed in our previous studies (Zhang et al., 2016; Ran et al., 2017; Zheng et al., 2017a, b, 2019b, 2020) and another study (Alvira et al., 2007). We found that 0.025, 0.05, and 0.1 g/kg dietary supplementation of RES induced hepatic immune response, and hence, those concentrations were also used in the present study. The used fundamental feed was a commercial diet purchased from Jiangsu Zhe Ya Food. Co., Ltd., China. RES was purchased from Sigma-Aldrich (St Louis, MO, United States). The feed was supplemented with RES at the following concentrations: 0.025, 0.05, and 0.1 g/kg. The ingredients are shown in Supplementary Table S1; the feed ingredients of each diet were powdered and mixed mechanically in a food mixer for approximately 40 min. Subsequently, water was added until a paste was obtained. The paste was then pelleted into 16-mm-diameter granular feed using a laboratory feed machine and air-dried at ambient temperature, dried at 60°C until being measured as 10% feed moisture, sieved, packed in plastic-lined bags, and stored at 4°C until further use. The experimental fish were fed once a day manually (4% of body weight as the recommended feed amount, with a total of 15.85 kg during the entire experimental period).

Prepared fish before sampling: The GIFT juveniles (*n* = 120) were assigned to 12 tanks (*n* = 10 per aquarium of 500 L, three tanks for each concentration in triplicate). The fish were divided into four different RES groups (*viz*., 0, 0.025, 0.05, and 0.1 g/kg) in triplicate. After the feeding trial, male fish (grow faster than females, weigh 41.2–50.9 g, and measure 10.5–11.4 cm, with an average growth rate of 1.0 g/d, Supplementary Table S2) were randomly selected for the exposure experiments.

### Fish Sampling

Sampled fish: Blood samples of male fish from different treatment groups were collected after fasting for 24 h on day 45, and named as follows: controls (Sample_Controls_1, 2, 3), 0.025 g/kg RES (Sample_0dian025_1, 2, 3, the total RES reached 3.3 mg per fish for the entire duration), 0.05 g/kg RES (Sample_0dian05_1, 2, 3, 6.6 mg/fish), and 0.1 g/kg RES (Sample_0dian1_1, 2, 3, 13.2 mg/fish). In each RES groups (three tanks), the fish blood was sampled (Ndong et al., 2007) for gene expression verification (*n* = 3), RNA-Seq (*n* = 3, blood of three fish from each tank was equimolarly mixed as one sequencing sample), and other parameters (*n* = 3). Particularly, samples for gene expression analysis were frozen in liquid nitrogen using Trizol reagent (Invitrogen, United States), and immediately stored at −80°C until further use.

### RNA-Seq Analysis, Functional Annotation, and Classification

RNA quantification and qualification were performed using the Qubit^®^ RNAAssay kit on Qubit^®^ 2.0 Fluorometer (Life Technologies, CA, United States) and Agilent Bioanalyzer 2100 system (Agilent Technologies, CA, United States). One microgram of RNA per sample was used as the input material for RNA sample preparations. Sequencing libraries were generated using the NEBNext^®^ Ultra^TM^ RNA Library Prep kit for Illumina (NEB, United States) following the manufacturer’s instructions. Finally, the sequencing libraries were sequenced on an Illumina Hiseq 2500 platform and paired-end reads were generated (Wang et al., 2015).

With respect to data analysis, raw data (raw reads) in the Fastq format were initially processed using in-house Perl scripts. In this step, adapters are usually trimmed, and then clean data (clean reads), reads containing poly-N, and low-quality reads (the score of base quality lower than Q30, Q30 means quality value (>30/total base) are obtained. All the downstream analyses were performed using the clean data with high quality (equal to or over Q30). These clean reads were then mapped to the reference genome sequence using Tophat2 tools (*O. niloticus* ASM185804v2). Gene function was annotated based on the following databases: Nr (NCBI non-redundant protein sequences); Nt (NCBI non-redundant nucleotide sequences); Pfam (protein family); KOG/COG (clusters of orthologous groups of proteins); Swiss-Prot (a manually annotated and reviewed protein sequence database); KO (KEGG ortholog database); and GO (gene ontology). Gene expression levels were estimated using the fragments per kilobase of transcript per million (FPKM) fragments mapped. The formula is shown below:

$$\text{FPKM}=\frac{\mathrm{cDNA}\,\mathrm{Fragments}}{\mathrm{Mapped}\,\mathrm{Fragments}\,\left(\mathrm{Millions}\right)\times \mathrm{Transcript}\,\mathrm{Length}\,\left(\mathrm{kb}\right)}$$

### Normalized Differential Expression Analysis and Comparison With the Increasing RES Concentration

Differential expression analysis of the four RES supplementation groups (0, 0.025, 0.05, and 0.1 g/kg) was performed using DESeq R package. The resulting *p*-values of DEGs were adjusted using Benjamini and Hochberg’s approach for controlling the false discovery rate. Genes with an adjusted *p*-value of <0.05 found by DESeq were considered differentially expressed.

The GO enrichment analysis of the DEGs was implemented in GO-seq R packages (Ashburner et al., 2000) based on Wallenius’ non-central hyper-geometric distribution (Young et al., 2010), which can adjust the gene length bias in DEGs. We used KOBAS (KEGG Orthology Based Annotation System, Xie et al., 2011) database to test the statistical enrichment of DEGs in the KEGG pathways (Kanehisa et al., 2008). We screened the significantly upregualted or downregulated genes by comparison among different RES concentration groups, and significant DEGs were enriched through comparison among the following six sets: 0.025 RES vs. Controls; 0.05 RES vs. Controls; 0.1 RES vs. Controls; 0.05 RES vs. 0.025 RES; 0.1 RES vs. 0.025 RES; 0.1 RES vs. 0.05 RES.

### Selected DEGs Associated With Mitochondrial Dysfunction

To further identify the mitochondria-related genes as the target genes, we screened the significant DEGs in the KEGG pathways associated with mitochondrial dysfunction, such as the terms of Alzheimer’s/Parkinson’s/Huntington’s disease and oxidative phosphorylation.

### qRT-PCR for Gene Expression Verification

The specificity test for RNA-Seq analysis was performed using the qRT-PCR method. RNA extraction, isolation, quality checking, and reverse transcription were conducted as previously described (Zheng et al., 2013). qRT-PCR was performed using the CFX96 thermocycler (Bio-Rad, United States) and SYBR Premix ExTaq II kit (TaKaRa, Japan). The qRT-PCRs were carried out in a reaction mixture of final volume 25 μL, containing 1 × SYBR *Premix Ex Taq^TM^*, 0.4 μM of each primer, and 2.5 μL of RT reaction solution. Cycling parameters were as follows: initial denaturation at 95°C for 30 s, followed by 40 cycles of denaturation at 95°C for 5 s, and annealing at 60°C for 30 s. Each individual sample was run in triplicate. A melt curve analysis was performed at the end of each PCR thermal profile to verify the specificity of each amplicon. Analysis of SYBR green I density and determination of threshold cycle (C_*t*_) values were carried out using CFX Manager software (Bio-Rad, United States). The efficiency (*E*) of each PCR was determined by the slope generated using 10-fold diluted cDNA series with five dilution points in triplicate. The equation was *E* = 10 ^(–1/slope)^.

The changes in gene expression were determined in the blood, and the sequence of qRT-PCR primers is shown in Supplementary Table S3 (Bustin et al., 2009). The gene expression profiles of nine genes (namely, *cox2*, *nad7*, *atpb*, *atpal*, *atpa*; *katna1*, *clpp*, *atp2b1*, and *gtpbpal*) were detected in the sampled blood tissues. However, genes associated with anti-oxidative system (*sod*, *cat*, *gpx*), apoptosis (*chk2*) and DNA damage (*rpa3*, Xu et al., 2020) have also been detected. β*-actin* was chosen as the reference gene as its expression remained constant among the experimental groups (Supplementary Table S4), and the cDNAs for the gene expression analysis were normalized with β*-actin*. The changes in the mRNA levels of these genes were calculated according to a previously described method (Livak and Schmittgen, 2001; Zheng et al., 2014).

### Content of MDA, GSH, ATP, and Activity of Ca^2+^-ATPase

The blood samples were homogenized in 0.86% cold physiological saline (1:9, w/v), and then centrifuged at 2,500 rpm at for 10 min 4°C; which used for the following detection: for ATP content analysis, the samples were centrifuged at 12,000 rpm for 10 min. The activity of Ca^2+^-ATPase, and the content of MDA (an indicator of increased oxidative stress), GSH (glutathione), and ATP in the supernatant were spectrophotometrically determined using commercially available kits (Nanjing Jiancheng Bioengineering Institute, China) according to the manufacturer’s instructions (including data analysis). The activity of Ca^2+^-ATPase was defined as the amount of inorganic phosphorus generated by 1 mg of protein per hour (expressed as U/mg prot). The content of MDA and GSH is expressed as μmol/g prot. The content of ATP is expressed as μmol/g protein.

### Comparative Analysis Between Blood and Hepatic Transcriptome

To screen for the different affected pathways and shared DEGs following RES administration, a comparison was made between blood transcriptome and previously reported hepatic transcriptome (Zheng et al., 2017a) in the 0.1 g/kg RES group on day 45. Simply, the sample method for hepatic transcriptome by Illumina Hiseq 2500 platform were as follows: all fish liver samples of different RES groups were collected at 45 d, control (the sample named T22, T23, T24), 0.025 g/kg RES (T05, T11, T17), 0.05 g/kg RES (T04, T10, T16), and 0.1 g/kg RES (T06, T12, T18). We first used the hepatic transcriptome set (*n* = 3, T06, T12, and T18 vs. T22, T23, and T24 marked as ”RES-vs-Control”) as the Control and the comparison of the 0dian1RES and control groups (*n* = 3, marked as ”0.1RES-vs-Con_1”) as the Case. We then compared the Control and Case using the previously reported hepatic transcriptome as the positive control.

### Statistics

Fish initial and final body weight, RNA counts, other parameters are expressed as mean ± SD, and they were tested for normal distribution (Kolmogorov Smirnov test) and homogeneity of variances (Levene’s test), prior to any additional analysis. Statistically difference was performed using one-way ANOVA and Duncan multiple range test with a significance level of *p* < 0.05.

## Results

### Functional Annotation and Classification

**FIGURE 1:**
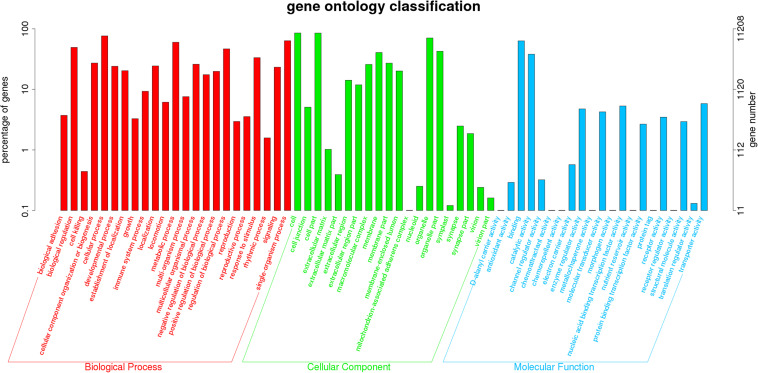
Comparative analysis on total gene expression regulation in three different concentrations of RES. A total of 11,208 unigenes were categorized into three functional categories: biological process, cellular component, and molecular function.

There were no statistically significant differences in the bodyweight or length in the exposure experiment (*P* > 0.05). No abnormal behavior or mortality occurred throughout the experimental period (*P* > 0.05). The GC content of the clean reads in the RES groups showed no significant difference when compared with the control groups (*P* > 0.05, Supplementary Table S5). A total of 11,208 unigenes (non-repeating sequence) were categorized into 64 sub-categories of three major categories, namely, biological process, cellular component, and molecular function (Figure 1). From the results of COG annotation, a total of 10,804 unigenes were successfully annotated into 25 categories (Supplementary Figure S1).

### Normalized Significant Differentially Expressed Genes (DEGs)

**FIGURE 2:**
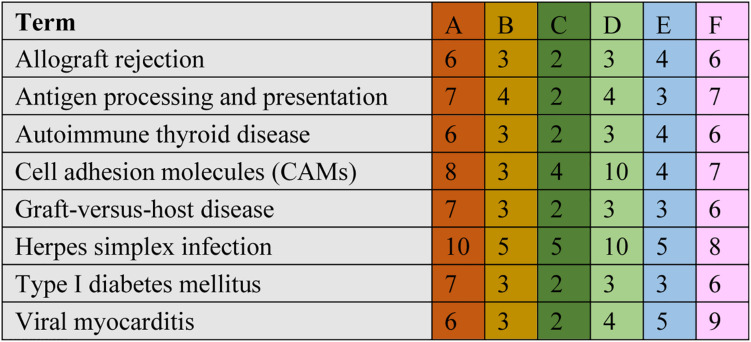
Comparative KEGG pathway analysis of DEGs in different RES groups. During the KEGG pathways classified from the significant DEGs, major of pathways have been selected. A stands for the comparison between 0.025 g/kg RES groups with the controls, B stands for the comparison between 0.05 g/kg RES groups with the controls, C stands for the comparison between 0.1 g/kg RES groups with the controls, D stands for the comparison between 0.1 with 0.025 g/kg RES groups, E stands for the comparison between 0.1 with 0.05 g/kg RES groups and F stands for the comparison between 0.1 g/kg RES groups with controls. The number in each column with different color means the statistical value of the significant DEGs in different RES groups when compared with the controls.

The significant DEGs for normalized gene expression among different RES supplementation groups were identified. In comparison with those of the control group, 918 (0.025 g/kg RES), 777 (0.05 g/kg RES), and 836 (0.1 g/kg RES) genes were detected as significant DEGs (Figure 2 and Supplementary Table S6), whose expression was not dose-dependent. The 0.05 and 0.1 g/kg RES groups presented 663 and 917 significant DEGs compared with those of the 0.025 g/kg RES groups, whereas the 0.1 g/kg RES groups presented 767 significant DEGs compared with those of the 0.05 g/kg RES groups. The highest amount of significant DEGs was obtained in those comparisons. A high percentage of the unigenes was mapped to the KEGG pathways associated with allograft rejection, antigen processing and presentation, autoimmune thyroid disease, cell adhesion molecules, graft-vs.-host disease, herpes simplex infection, type I diabetes mellitus, and viral myocarditis pathways (Figure 2). The results from KEGG pathway analysis revealed that the genes were enriched to 366 KEGG pathways, of which 51 pathways (Figure 2) were related to immune functions (therein 22 pathways are shown in Table 1, *P* < 0.05).

**TABLE 1:** The significant KEGG pathways gathered according to the comparison between different concentrations of RES addition.

Pathway term	Pathway_id	List hits	*P*-value
Herpes simplex infection	ko05168	43	0.0143
Epstein-Barr virus infection	ko05169	38	0.0088
Cell adhesion molecules (CAMs)	ko04514	36	0.0046
Viral carcinogenesis	ko05203	29	0.0080
Phagosome	ko04145	25	0.0030
Viral myocarditis	ko05416	25	0.0002
Antigen processing and presentation	ko04612	23	0.0084
Alzheimer’s disease	ko05010	23	0.0015
Type I diabetes mellitus	ko04940	21	0.0033
Autoimmune thyroid disease	ko05320	21	0.0003
Allograft rejection	ko05330	21	0.0004
Graft-vs.-host disease	ko05332	21	0.0018
Parkinson’s disease	ko05012	20	0.0007
Natural killer cell mediated cytotoxicity	ko04650	18	0.0066
Long-term depression	ko04730	17	0.0050
Huntington’s disease	ko05016	15	0.0032
Carbon metabolism	ko01200	14	0.0007
NOD-like receptor signaling pathway	ko04621	13	0.0001
Biosynthesis of amino acids	ko01230	12	0.0026
Influenza A	ko05164	12	0.0029
Oxidative phosphorylation	ko00190	11	0.0018
Endocytosis	ko04144	11	0.0343

**T*hree hundred sixty-six *KEGG pathways have been matched in the transcriptional analysis, of which 51 pathways were related to immune functions. T*wenty-two *pathways (list hits > 10, P < 0.05) have been revealed.**

### Significant DEGs Among Different RES Groups

A comparison between the high RES supplementation groups and low RES supplementation groups (including the control group), revealed that no significant DEGs were enriched among the six groups (0.025 RES vs. Controls; 0.05 RES vs. Controls; 0.1 RES vs. Controls; 0.05 vs. 0.025 RES; 0.1 vs. 0.025 RES, and 0.1 vs. 0.05 RES, *P* > 0.05).

**FIGURE 3:**
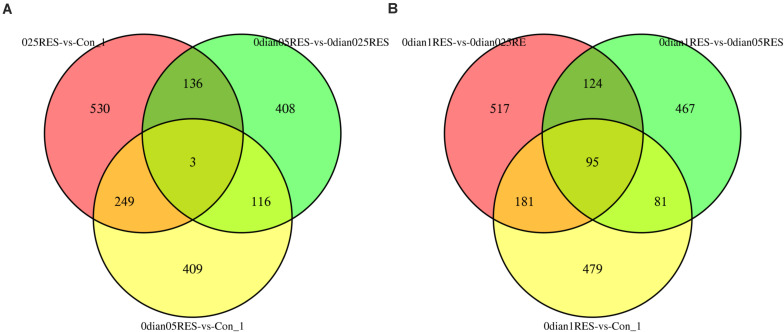
Comparative analysis venn map on gene expression regulation in four different concentrations of RES. **(A)** Red area stands for the comparison between 0.025 g/kg RES groups with the controls, green area reveals the comparison between 0.025 and 0.05 g/kg RES, and yellow area means the comparison between 0.05 g/kg RES groups with the controls. **(B)** Red area stands for the comparison between 0.025 with 0.1 g/kg RES groups, green area means the comparison between 0.05 and 0.1 g/kg RES, and yellow area reveals the comparison between 0.1 g/kg RES with the controls.

A comparison (0.025 vs. Con; 0.05 vs. Con; 0.05 vs. 0.025) among the three groups (the controls, 0.025 RES and 0.05 RES groups) revealed that, only three (CL1317/*katna1*, comp48583*/cox2*, CL8/*nad7*) shared significant DEGs were enriched. Considering the significant effect in the 0.1 g/kg RES groups at the genome level, another comparison between the highest RES group (0.1 g/kg) and the other three groups (the controls, 0.025 RES and 0.05 RES groups, 0.1 vs. 0.05/0.025/Con) showed that 95 shared significant DEGs were enriched (Figure 3). Ninety-five shared significant DEGs were classified into 13 different terms (Supplementary Table S7), and some were related to the ATP synthase activity (GO: 0046933, 0015986, 0009055, and 0005524), function of chloroplasts (GO: 0016168, 0009535, and 0009507), and others were associated with translation (GO: 0018298, 0003735, 0006412, 0006310, 0003723, and 0003964).

**FIGURE 4:**
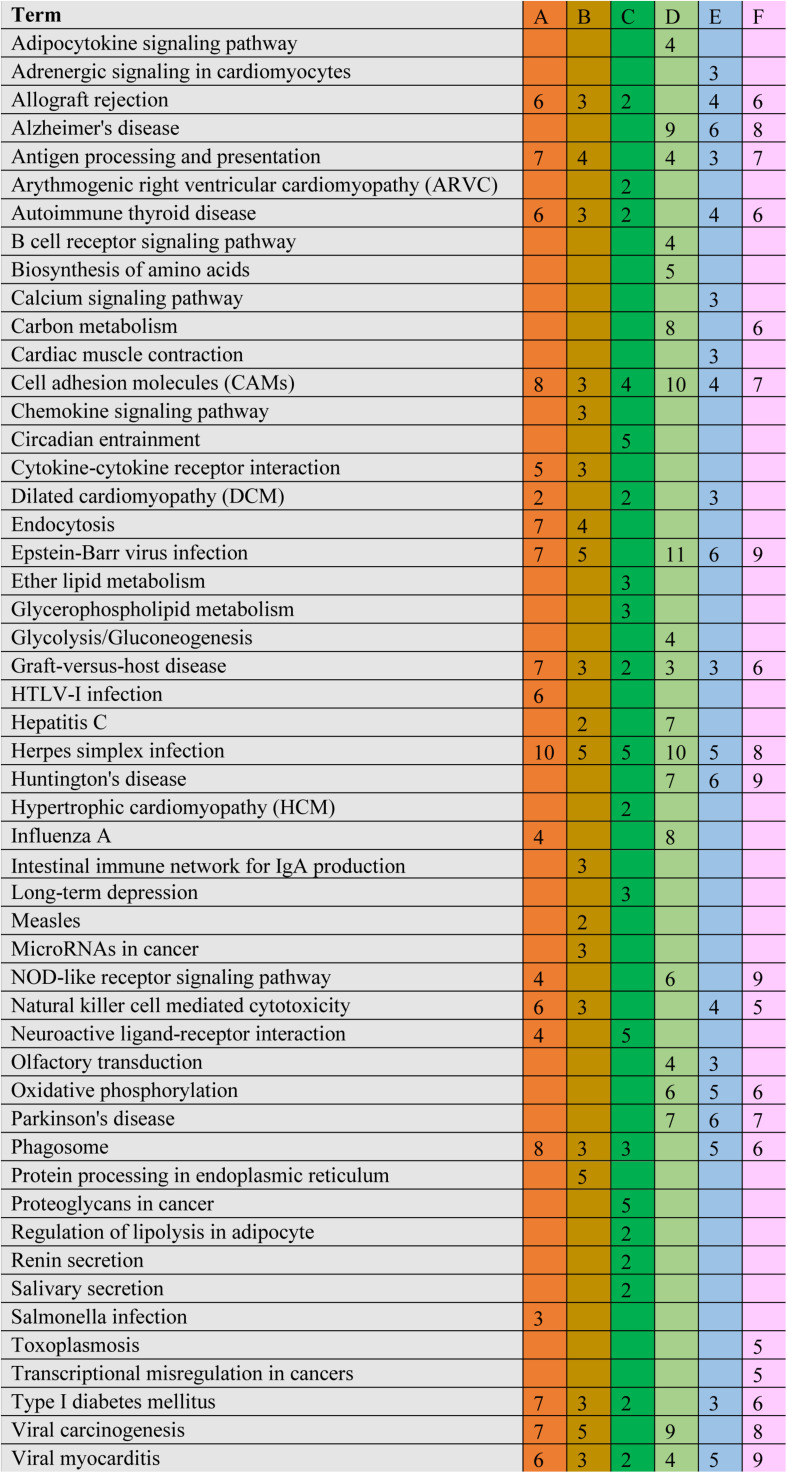
Cluster of orthologous groups (COG) classification associated with mitochondrial dysfunction. When we compared with different groups, we found ranging from 6 to 10 significant DEGs in the comparisons, which may be classified into allograft rejection, antigen processing and presentation, autoimmune thyroid disease, cell adhesion molecules (CAMs), graft-vs.-host disease, herpes simplex infection, type I diabetes mellitus, and viral myocarditis. The annotation was the same to Figure 2.

A comparison between the 0.025 RES and control groups revealed 6–10 significant DEGs, which might be classified into allograft rejection, antigen processing and presentation, autoimmune thyroid disease, cell adhesion molecules (CAMs), graft-vs.-host disease, herpes simplex infection, type I diabetes mellitus, and viral myocarditis (Figure 4).

### Selected DEGs Associated With Mitochondrial Dysfunction

To identify DEGs, we chose some genes (the primers, symbols, names and GenBank IDs are presented in Supplementary Table S5) to verify their specificity for the transcriptional analysis (Figure 4). The four significant KEGG pathways were Alzheimer’s/Parkinson’s/Huntington’s disease, and oxidative phosphorylation (Table 2). On day 45, some genes related to ATP synthase or mitochondrial dysfunction, including *katna1*, and *cox2* were significantly upregulated in the 0.1 g/kg RES group in comparison with those in the other three treatment groups (*P* < 0.05, Supplementary Table S8). *nad7* was significantly downregulated in the 0.1 g/kg RES group compared with that in the other three treatments (*P* < 0.05).

**TABLE 2:** The assembled significant KEGG pathways.

Number	ID	Term	Enrichment_Score
1	ko05012	Parkinson’s disease	12.97
2	ko05010	Alzheimer’s disease	11.30
3	ko05016	Huntington’s disease	9.95
4	ko00190	Oxidative phosphorylation	12.00

*Enrichment score = (m/n)/(M/N): “N” was the number of GO annotated genes in all genes; “n” was the number of GO annotated genes in DEGs in “N.” “M” was the number of genes that are annotated as a specific GO term in all genes, and “m” is annotated as the number of DEGs in a specific GO term.*

Four genes associated with the same KEGG pathway term (Alzheimer’s/Parkinson’s/Huntington’s disease and oxidative phosphorylation), including *atpb*, *atpal*, *atpa*, and *clpp* showed the increased expressions in the 0.025 g/kg RES group and decreased expressions in the 0.05 g/kg RES group when compared with those in the control groups, whereas nearly no expression was observed in the 0.1 g/kg RES group.

Two genes, *atp2b1* (hydrolysis of ATP) and *gtpbpal* (regulation of circadian mRNA stability) were initially expressed only in the 0.1 g/kg RES group in comparison with those in the controls, 0.025 g/kg RES, and 0.05 g/kg RES group.

### qRT-PCR Verification for Transcriptome Analysis

**FIGURE 5:**
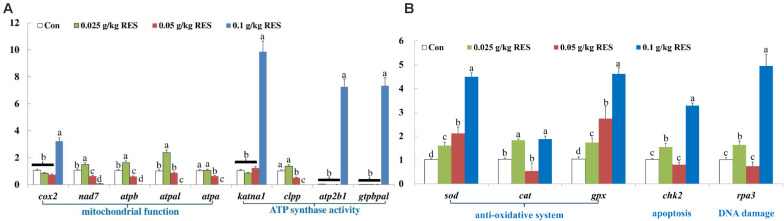
**(A)** Genes related to mitochondrial function and ATP synthase activity. **(B)** Genes related to anti-oxidative system, apoptosis and DNA damage. qRT-PCR verification. The selected nine DEGs in different KEGG pathways associated with mitochondria at 45 days (*n* = 9) were as follow: *cox2*, cytochrome c isoform X2; *nad7*, NADH dehydrogenase [ubiquinone] iron-sulfur protein 2 mitochondrial; *atpb*, ATP synthase subunit beta, mitochondrial; *atpal*, ATP synthase subunit alpha, mitochondrial-like; *atpa*, ATP synthase subunit alpha, mitochondrial; *katna1*, katanin p60 ATPase-containing subunit A1 isoform X1; *clpp*, ATP-dependent Clp protease proteolytic subunit, mitochondrial; *atp2b1*, plasma membrane calcium-transporting ATPase 1-like; *gtpbpal*, GTP-binding protein A-like. Different lowercase letters represent significant differences between values at *P* < 0.05.

The DEGs were verified by qRT-PCR, which showed good correlation with the mRNA-Seq results (Supplementary Table S9). The expression of *cox2*, *katna1*, *atp2b1*, and *gtpbpal* was significantly increased in the 0.1 g/kg RES group compared with that in the other groups (*n* = 9, *P* < 0.05, Figure 5A). The expression of *nad7*, *atpb* and *atpal* was significantly increased in the 0.025 g/kg RES group (*P* < 0.05), whereas that of *atpa* and *clpp* showed no significant differences between the 0.025 g/kg RES and control groups (*P* > 0.05); however, the expression of these genes was significantly increased in the 0.025 g/kg RES group compared with that in the 0.05 and 0.1 g/kg RES groups (*P* < 0.05). *sod* and *gpx* was significantly increased across the concentration, while *cat*, *chk2* and *rpa3* revealed “U” shaped expression pattern (lowest in the middle RES concentration, *P* < 0.05) (Figure 5B), with no significant difference in the 0.05 g/kg RES group when compared with that in the control group (*P* > 0.05).

### Contents of MDA, GSH, ATP, and Activity of Ca^2+^-ATPase

**FIGURE 6:**
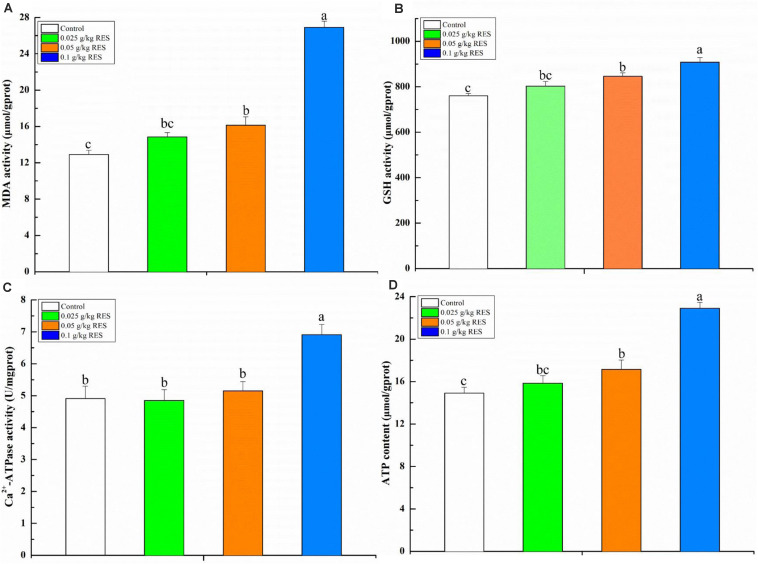
Blood antioxidative parameter concentrations and ATP content by RES supplementation **(A)** MDA, **(B)** GSH, **(C)** Ca^2+^-ATPase, **(D)** ATP content. Different lowercase letters stand for significant change with a significance level of *p* < 0.05.

To verify the product of DEGs associated with mitochondrial dysfunction, the blood contents of MDA, GSH, ATP, and activity of Ca^2+^-ATPase were determined. The blood MDA, GSH, and ATP content (linearity coefficient ranged from 0.9993 to 0.9998) was significantly increased in the 0.1 g/kg RES group compared with that in the 0.05 g/kg RES and control groups (*P* < 0.05), whereas no significant difference was observed in the 0.025 g/kg RES group compared with that in the 0.05 g/kg RES and control groups (*n* = 9, *P* > 0.05, Figure 6). The blood Ca^2+^-ATPase activity was significantly increased in the 0.1 g/kg RES group compared with that in other groups (*P* < 0.05).

### Comparative Analysis Between Blood Transcriptome and Hepatic Transcriptome

**FIGURE 7:**
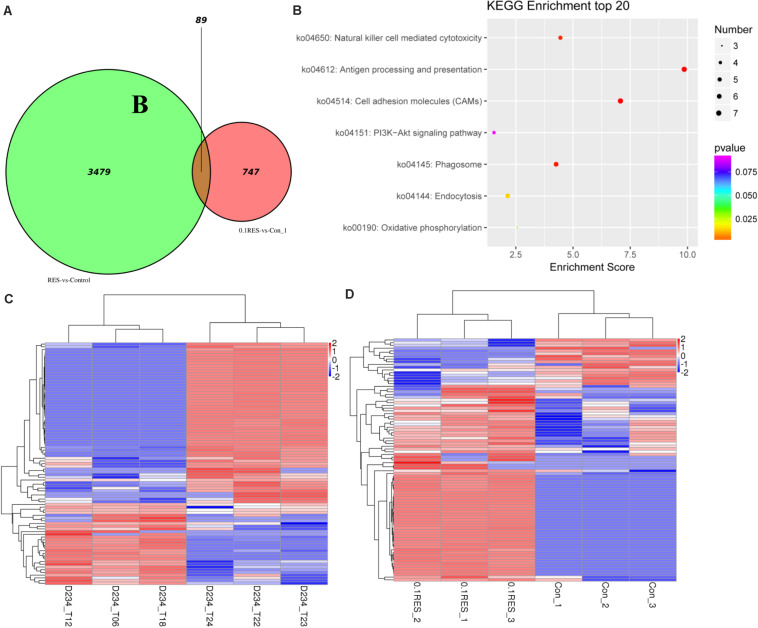
Comparative analysis venn, hot map, and KEGG enriched pathways. The comparison based on blood (this study, the comparison between 0.1 g/kg RES groups and the controls, named 0.1RES vs. Con_1) and hepatic (our previous work, named RES vs. Control) transcriptome. **(A)**, venn map of shared 89 DGEs, the value 3,479 and 747 means hepatic and blood specific DGEs in the 0.1 g/kg RES groups when compared with the controls, while the value 89 stands for the shared specific DGEs. KEGG enriched pathways **(B)** of 89 shared DGEs in the hepatic and blood transcriptome revealed. The hot map of 89 shared DGEs in the hepatic **(C)** and blood **(D)** transcriptome.

We screened for differences between the blood transcriptome and previously reported hepatic transcriptome (Zheng et al., 2017a) in the 0.1 g/kg RES group on day 45. The results demonstrated that 3,479 and 747 DEGs were enriched as specifically expressed genes (expressed/not expressed when compared with the corresponding controls) (Figure 7A). In the hepatic and blood transcriptomes, 89 shared DEGs were mainly enriched in antigen processing and presentation, cell adhesion molecules, and phagosome pathways (Figure 7B). Among those 89 DEGs, 30 were upregulated in the hepatic transcriptome (Figure 7C), whereas 72 were upregulated in the blood transcriptome (Figure 7D). Compared with those in the hepatic transcriptome, 67 and 22 DEGs were upregulated and downregulated in the blood transcriptome, respectively (Supplementary Table S10).

## Discussion

Currently, the RES dietary supplements are commercially available and marketed as anti-inflammatory “nutraceuticals.” When used as a feed additive for GIFT in our previous studies, we found that RES exerted important modulatory effects on the inflammatory responses in the liver and blood of tilapia (Zheng et al., 2017b). Some morphological changes, such as necrosis, apoptosis (Zhu et al., 2017), hyperemia, and fibrosis of liver cells immediately occurred after the physiological regulation (Zheng et al., 2017b). Our recent study indicated that oxidative status might be the effective response pathway involved in the immune system in fish supplemented with RES as an immunopotentiator, based on hepatic transcriptome analysis using the RNA-Seq method (Zheng et al., 2017a), which has been confirmed by qRT-PCR verification of anti-oxidative system related genes (*sod*, *cat*, and *gpx*) in serum of the present study. During the inflammation process, cytokine production in the blood of tilapia has been observed; however, this phenomenon has not been revealed in the blood transcriptome analysis. But in other affected pathways, *chk2* in *p*53 pathway, *rpa3* in DNA repair pathway significantly increased in 0.025 and 0.1 g/kg RES in the present study. In the present study, the blood transcriptome of GIFT was characterized to identify the mitochondria-related genes and biological processes involved in the innate response, which was the same to the previous study (affected the innate immunity of fish, Zhang et al., 2014). Could the increased cytokine production alert the serial fluctuated change in liver? In the comparison between hepatic (previous data) and blood transcriptomes, 89 shared DEGs were enriched in antigen processing and presentation (Bonifaz et al., 2015; Matheoud et al., 2016), cell adhesion molecules (associated with phosphorylation and apoptosis (Gledhill et al., 2007; Lin et al., 2009) via the Sirt1-PGC1α-PPARα pathway (Milton-Laskibar et al., 2018), and the phagosome (Rubinsztein et al., 2011) pathways. However, 3479 and 747 DEGs were enriched as specifically expressed genes in liver and blood, and it hinted that RES aroused different response in blood (differ from liver), but it may exist some crosstalk based on the shared pathways (like circulation axis, Xu et al., 2018; Zhou et al., 2019).

Actually no reports focused on the pathological features of Alzheimer’s Disease in fish, but a lot of studies used zebrafish as the model for discussing how to care with neurological disorders, or nervous system diseases. Lots of studies demonstrated RES affected neurological disorder or disease in different fish species, like zebrafish (Yang et al., 2017; Peñalver et al., 2018). RES has also been shown to potentially target several mitochondrial metabolic pathways (fatty acid β-oxidation and respiratory chain) or oxidative phosphorylation pathway (Bastin and Djouadi, 2016; Jardim et al., 2017), suggesting that RES has a therapeutic effect associated with a variety of mitochondrial dysfunctions. However, another study revealed that RES has no effect in patients with Alzheimer’s disease (Manczak et al., 2010). RES treatment attenuated rotenone-induced Parkinson’s like behavioral alterations, oxidative stress, and mitochondrial dysfunction in rats (Palle and Neerati, 2018). It has been demonstrated that RES alleviated LPS-induced apoptosis of hippocampal cells by increasing mitochondrial superoxide production and decreasing mitochondrial membrane potential and ATP production in the hippocampus (Chen et al., 2017).

Regardless of the data obtained by transcriptome analysis or qRT-PCR verification, the present study showed that the expression of *nad7* decreased following 0.05 and 0.1 g/kg RES supplementation, whereas its expression increased in the 0.025 g/kg RES group. The expression of *atpb*, *atpal*, and *atpa* in the lower supplementation RES groups (0, 0.025, and 0.05 g/kg) was higher than that in the higher RES supplementation group (0.1 g/kg). Interestingly, the four significant KEGG pathways enriched by the above genes were related with Alzheimer’s/Parkinson’s/Huntington’s disease and oxidative phosphorylation, and the expression of those genes was significantly altered in the present study. With respect to the expression product of significant DGEs, malondialdehyde (MDA, an indicator of increased oxidative stress) and glutathione (GSH) contents was further determined, and the results revealed that RES exerted anti-oxidative effect by increasing the blood content of MDA and GSH in the 0.1 g/kg RES group. Our previous study results showed that *dnah7* × *1* (dynein heavy chain 7, axonemal isoform X1, ATPase activity, and functional maintenance of microtubules), *hsp90a* (heat shock protein HSP 90-alpha-like, oxidative stress pathway), and *nmrk2* (nicotinamide riboside kinase 2-like, energy production) were up-regulated following RES supplementation (Zheng et al., 2017a). The present study demonstrated that RES not only affects oxidative stress but also mitochondrial dysfunction.

Studies have showed that the production of ATP in cells positively and negatively correlated with RES concentration and that the mitochondrial membrane potential was lost with increased concentration of RES. The ATP content was significantly increased among the RES concentration groups. RES treatment was effective in enhancing ATP generation (Abe et al., 2017), GSH activity and cytochrome c release under mitochondrial injury in rat hepatocytes (Al Maruf et al., 2018). RES increased the expression of rat *cox2* (cytochrome c); the expression of *cox2* was also increased following RES supplementation in the present study, which demonstrates that RES exerts body-fat lowering effect (Alberdi et al., 2013). The relationship between cytochrome c release with its associated pathway (caspase/NF-κB) and RES treatment has not been determined (Fu et al., 2013; Lin et al., 2014).

Mitochondria are the principal regulators of cellular function and metabolism via the production of ATP for energy homeostasis, maintenance of calcium homeostasis, and regulation of apoptosis for fueling the electron transport chain. Mitochondria releases signal to modulate innate immunity and systemic inflammatory responses and could consequently promote inflammation during aging (Hill and Van Remmen, 2014). The present study showed that the expression of *katna1* was significantly increased in the 0.1 g/kg RES group, whereas *clpp* and *gtpbpal* were slightly and highly expressed in the 0.1 g/kg RES group, respectively. However, *atp2b1* (plasma membrane calcium-transporting ATPase 1-like) was highly expressed in the 0.1 g/kg RES groups, and blood Ca^2+^-ATPase activity was significantly increased in the 0.1 g/kg RES group when compared with the controls. The intracellular calcium release and calcium influx decreased gradually with the increase of RES concentration (Cao et al., 2018). It has been reported that RES might affect calcium release and influx, thereby inducing changes in the mitochondrial electron transfer chain by generating higher amounts of superoxide anion (Costa et al., 2016).

Both decreased nutritional status and environmental factor influence the physiological tolerance and health of fish populations (Haller et al., 2015). A recent study demonstrated that the elevated body temperatures associated with endothermy might lead to a compensatory decrease in mitochondrial ROS production relative to respiratory capacity when compared with that in marine fish (Wiens et al., 2017). However, feed restriction and environmental factors (salinity level and exposure time) exerted significant effects on Na^+^/K^+^-ATPase and morphology of gill mitochondria-rich cells in fish (Haller et al., 2015). Our previous study showed that the supplementation of RES might disturb the cytokine pathway via the inflammatory response (Zheng et al., 2017a), and higher RES supplementation might lead to mitochondrial dysfunction or hepatic apoptosis, which might result in organ impairment (Zheng et al., 2017a). In that study, the feed additive limit of RES was 0.025 g/kg, which was obtained based on the results of intestinal microbiota sequencing (increased beneficial and decreased harmful bacteria) (Zheng et al., 2018, 2019b). A selective approach for immune enhancement could be floating bed cultivation with medicine food homology plant (e.g., *Polygonum cuspidatum*); one goal was to change gut contents and regulate microenvironment balance by absorbing active constituents (Zheng et al., 2019a, 2020), another was to release allelochemicals against fish pathogenic bacteria through long-time duration. Field fish farmers can easily accept the use of medical herbs instead of vegetables in order to earn more money (as food or by selling to plant extraction companies). The data of the present study demonstrated that RES supplementation might result in preventing mitochondrial dysfunction via energy synthesis or electron transfer chain. Whereas, the expression of *clpp* (longevity regulating pathway) increased only in the 0.025 g/kg RES group (the appropriate RES level for tilapia) compared with that in the other groups. It has been demonstrated that low RES supplementation might have anti-aging effects by preventing the inflammatory response or eliminating reactive oxygen species.

Some kinds of immunomodulators (e.g., fulvic acid and probiotics) have been used in fish feed, while RES is recommended as an anti-inflammatory dietary supplement for domestic animals and is reported to downregulate pro-inflammatory cytokine production (Liu et al., 2015). RES exaction has been used as a feed additive from the leaf of *P. cuspidatum* (*Huzhang* in Chinese). However, RES significantly normalized the mitochondrial inner membrane polarization in the axons through the adenosine monophosphate-activated protein kinase/PGC-1α pathway (Gledhill et al., 2007; Rodgers et al., 2008; Lin et al., 2009; Rubinsztein et al., 2011; Roy Chowdhury et al., 2012; Yuan et al., 2012; Milton-Laskibar et al., 2018). The anti-apoptotic effect of RES was independent of the stimulation of silent information regulator of transcript 1 (SIRT1) and dependent on its anti-oxidant properties (Simó-Mirabet et al., 2018). In cattle, the beneficial effects of dietary RES might derive in part by preventing mitochondrial ATP synthesis in tumor cells, thereby inducing apoptosis (Gledhill et al., 2007; Lin et al., 2009) via the Sirt1-PGC1α-PPARα pathway (Milton-Laskibar et al., 2018). However, it has been further confirmed in teleosts through the NAD^+^/NADH ratio (Zhang et al., 2017), phosphorylation (Shi et al., 2018) and acetylation (Schirmer et al., 2012) in post-transcriptional regulation levels. Though the expression of genes related with mitochondrial dysfunction and ATP synthese activity changed, future studies should be conducted to determine the actual pathways involved.

## Conclusion

In the present study, 51 KEGG pathways were enriched among different RES supplementation groups, 3 and 95 shared significant DEGs were enriched between the RES supplementation and control groups, and among the different RES concentration groups (0.025, 0.05, and 0.1 g/kg RES). Among these 95 significant DEGs, 8 COG classification associated with mitochondrial dysfunction and four KEGG pathways associated with mitochondria were enriched. The final gene verification supported the results that obtained from the blood transcriptome. *cox2*, *katna1 atp2b1*, and *gtpbpal* showed the highest expression in the 0.1 g/kg RES group, while *nad7*, *atpb*, *atpal*, *atpa*, and *clpp* revealed a dose dependent expression following RES supplementation. Such transcriptional changes corresponds with the increased ATP-related enzymes in the 0.05 and 0.1 g/kg RES groups. Genes associated with anti-oxidative system (*sod*, *cat*, *gpx*), apoptosis (*chk2*) and DNA damage (*rpa3*) significantly increased in the 0.025 and 0.1 g/kg RES groups. The present study suggested RES supplementation might affected mitochondrial dysfunction associated with disease resistance, energy synthesis.

## Data Availability Statement

The datasets generated for this study can be found in the online repositories. The names of the repository/repositories and accession number(s) can be found in the article/Supplementary Material.

## Ethics Statement

The animal study was reviewed and approved by the Ministry of Science and Technology of the People’s Republic of China (Approval ID: 2011AA1004020012). Written informed consent was obtained from the owners for the participation of their animals in this study.

## Author Contributions

YZ and JC conceived and designed the experiments. YZ analyzed the data, contributed to figures preparation, and prepared and wrote the manuscript. LQ, WW, and GH contributed to the reagents, materials, and analysis tools. All authors reviewed the manuscript.

## Conflict of Interest

The authors declare that the research was conducted in the absence of any commercial or financial relationships that could be construed as a potential conflict of interest.

## Abbreviations

BLAST: basic local alignment search tool
DEGs: differentially expressed genes
FPKM: fragments per kilobase of transcript per million
GIFT: genetically improved farmed tilapia
GSH: glutathione
LSD: least significant difference test
MDA: malondialdehyde
RES: resveratrol
SD: standard deviation.
