# Supplementary material for: Transcriptome Analysis of Juvenile Tilapia (Oreochromis niloticus) Blood, Fed With Different Concentrations of Resveratrol
Source: Front Physiol. 2020 Dec 9;11:600730. doi: 10.3389/fphys.2020.600730 (PMC7755862; doi:10.3389/fphys.2020.600730)
Supplement: Supplementary file 1 [file Table_1.docx]

Table 1 The significant KEGG pathways gathered according to the comparison between different concentrations of RES addition. 366 KEGG pathways have been matched in the transcriptional analysis, of which 51 pathways were related to immune functions. 22 pathways (list hits >10, *P*-value<0.05) have been revealed.

| **#pathway term** | **pathway_id** | **List Hits** | ***P*-value** |
| --- | --- | --- | --- |
| Herpes simplex infection | ko05168 | 43 | 0.0143 |
| Epstein-Barr virus infection | ko05169 | 38 | 0.0088 |
| Cell adhesion molecules (CAMs) | ko04514 | 36 | 0.0046 |
| Viral carcinogenesis | ko05203 | 29 | 0.0080 |
| Phagosome | ko04145 | 25 | 0.0030 |
| Viral myocarditis | ko05416 | 25 | 0.0002 |
| Antigen processing and presentation | ko04612 | 23 | 0.0084 |
| Alzheimer's disease | ko05010 | 23 | 0.0015 |
| Type I diabetes mellitus | ko04940 | 21 | 0.0033 |
| Autoimmune thyroid disease | ko05320 | 21 | 0.0003 |
| Allograft rejection | ko05330 | 21 | 0.0004 |
| Graft-versus-host disease | ko05332 | 21 | 0.0018 |
| Parkinson's disease | ko05012 | 20 | 0.0007 |
| Natural killer cell mediated cytotoxicity | ko04650 | 18 | 0.0066 |
| Long-term depression | ko04730 | 17 | 0.0050 |
| Huntington's disease | ko05016 | 15 | 0.0032 |
| Carbon metabolism | ko01200 | 14 | 0.0007 |
| NOD-like receptor signaling pathway | ko04621 | 13 | 0.0001 |
| Biosynthesis of amino acids | ko01230 | 12 | 0.0026 |
| Influenza A | ko05164 | 12 | 0.0029 |
| Oxidative phosphorylation | ko00190 | 11 | 0.0018 |
| Endocytosis | ko04144 | 11 | 0.0343 |
